# Pediatric Myocarditis Management With Dual Immunotherapy: A Case Report Highlighting IVIG and Methylprednisolone Efficacy

**DOI:** 10.7759/cureus.86073

**Published:** 2025-06-15

**Authors:** Ahmad I Owdat, Ahmad Alhajieh, Rashed Alsheab, Ammar ALghsoon, Sura Alhunaifat, Noor Alrawajfeh, Rawia Alfarajat

**Affiliations:** 1 Pediatrics, Jordanian Royal Medical Services, Irbid, JOR; 2 Pediatrics, Jordanian Royal Medical Services, Zarqa, JOR; 3 Pediatrics, Jordanian Royal Medical Services, Amman, JOR; 4 General Medicine, Al-Balqa' Applied University, Amman, JOR; 5 General Medicine, Jordanian Ministry of Health, Amman, JOR

**Keywords:** acute myocarditis in children, corticosteroids in myocarditis, dual immunotherapy, ivig (intravenous immunoglobulin), rescue therapy for myocarditis

## Abstract

Myocarditis is an acute inflammation of the cardiac muscle cells, resulting in variable degrees of myocardial dysfunction, typically as a result of a viral infection. Clinical symptoms can vary widely depending on the severity of the disease. Even though there are several therapeutic approaches for pediatric myocarditis, the treatment is usually dominated by corticosteroids. Some form of immunomodulatory treatment, most notably intravenous immunoglobulin (IVIG), has been implemented in practice but their effectiveness is controversial and needs more research.

We document the case of a 12-month-old female infant who presented with a three-day history of fever, tachypnea, lethargy, and poor feeding. A workup including laboratory tests, chest X-ray, electrocardiogram, and echocardiogram revealed cardiac dysfunction supporting the diagnosis of acute myocarditis. She received IVIG and methylprednisolone, which resulted in marked clinical improvement, although some degree of cardiac dysfunction persisted. At the time of reporting, she remained under ongoing follow-up care. This case underlines the possible impact of prescribing IVIG with methylprednisolone on children with acute pediatric myocarditis, particularly in resource-limited settings where access to advanced treatment options may be restricted.

## Introduction

Myocarditis is a challenging and heterogeneous cardiac condition in children, primarily due to its variable etiology, non-specific clinical presentation, and the complexity of diagnosis and treatment. Myocarditis can be classified into acute, fulminant, chronic active, and persistent chronic, each requiring different treatment tactics [1]. Acute myocarditis is a rare, yet highly dangerous phenomenon in children defined as the inflammation of cardiac myocytes. According to various studies, myocarditis has an incidence of about 0.02%-0.05% in pediatric cases [2].

The majority of pediatric myocarditis cases are believed to be viral in origin, with common causative agents including Coxsackievirus B, parvovirus B19, adenovirus, enteroviruses, influenza virus, Epstein-Barr virus, and cytomegalovirus. Among these, Coxsackievirus B is particularly well-established as a frequent cause in children. Non-infectious causes, though less frequently highlighted in clinical discussions, are equally important and include bacterial and protozoal infections, hypersensitivity reactions to drugs or toxins, and autoimmune disorders such as systemic lupus erythematosus and sarcoidosis. Recognizing these varied etiologies is essential, as they may require distinct management strategies.

Diagnosis remains a significant challenge due to the non-specific nature of symptoms, which often include chest pain, dyspnea, palpitations, fatigue, and syncope, or may mimic respiratory infections. It may even lead to cardiogenic shock alongside brady- and tachyarrhythmias, with conduction imbalances [3]. Importantly, myocarditis should be considered in the differential diagnosis of any child presenting with unexplained respiratory distress or hemodynamic instability [4].

Historically, the definitive diagnosis of myocarditis relied on endomyocardial biopsy (EMB), assessed using the Dallas criteria, which identify myocardial inflammation and necrosis histologically [5]. However, the invasive nature of EMB, associated procedural risks, and high false-negative rates due to sampling error (estimated >25%) [6,7] have shifted clinical preference toward non-invasive modalities, particularly cardiac magnetic resonance imaging (CMR). CMR provides valuable tissue characterization and is now widely accepted as a key diagnostic tool, especially when interpreted using the 2018 revised Lake Louise criteria [8].

Diagnosis is typically supported by a combination of clinical history, laboratory tests (e.g., elevated troponin, CK-MB, inflammatory markers), electrocardiogram changes, echocardiography, and imaging findings. Despite advancements in diagnostic tools, confirming the etiology and optimal therapeutic approach remains difficult, especially in resource-limited settings where access to CMR or biopsy may be constrained.

For immune-mediated myocarditis, corticosteroids remain a cornerstone of therapy due to their anti-inflammatory properties. However, the use of other immunosuppressive agents, such as IVIG, remains a topic of ongoing debate. While some clinical studies and randomized controlled trials suggest that IVIG may improve ventricular function in children with acute viral myocarditis, the evidence remains inconclusive and does not yet support universal adoption. IVIG has both pro- and anti-inflammatory effects; in the context of myocarditis, it is hypothesized to mitigate myocardial injury by neutralizing autoantibodies, reducing cytokine release, and modulating T-cell responses - mechanisms relevant to post-viral or autoimmune-mediated cardiac inflammation [1].

The overall prognosis for pediatric myocarditis is fair, especially concerning their survival, with estimates of reaching 80%. In the first stages, the mortality can lie between 7% and 15% [9]. The absence of a universally accepted definition of myocarditis continues to hinder progress in developing targeted therapies [1].

Intravenous immunoglobulin (IVIG) is derived from pooled human plasma and contains immunoglobulin (Ig) G of all subclasses and allotypes but only minimal amounts of IgM and IgA, and it has shown to have antiviral, immunomodulatory effects and immunosuppressant properties. Studies have shown that IgG and polyvalent intravenous IgG, IgA, and IgM exert proinflammatory effects, including the activation of immune cells, the activation of the complement system, and the opsonization of infective agents. They also have anti-inflammatory effects, which comprise the neutralization of bacterial and other toxins, degradation products, and an excess of complement factors and cytokines, which help balance the proinflammatory process [10]. IVIG can modulate the inflammatory and immune response without major side effects. Thus, if there is an ongoing infection, a post-infectious inflammatory reaction, or a non-infectious process, IVIG can be efficacious [11].

We report a case of a 12-month-old female infant with a prior history of upper respiratory tract infection who developed respiratory distress and disturbed vitals. Initial echocardiogram and laboratory findings confirmed acute heart failure with reduced ejection fraction and fractional shortening. The patient was managed with early use of IVIG and methylprednisolone. We discuss the clinical course, safety considerations, and observed short-term benefits of this dual immunotherapy, while also emphasizing the need for further research, particularly in resource-limited environments where diagnostic and treatment options are often constrained.

## Case presentation

A 12-month-old previously healthy female infant, fully vaccinated according to the Jordanian national immunization program, presented with a three-day history of fever, lethargy, and dyspnea. The patient had recently experienced an upper respiratory tract infection, which was managed at home with antipyretics and antihistamines. Over the preceding 24 hours, her parents noted a decline in physical activity and poor oral intake.

On physical examination, the infant appeared acutely ill and exhibited signs of respiratory distress, including tachypnea, nasal flaring, and suprasternal retractions. Vital signs were as follows: heart rate of 164 beats per minute, blood pressure of 90/60 mmHg, respiratory rate of 28 breaths per minute, and oxygen saturation of 92% on room air. The axillary temperature was 38.3°C. Cardiac examination revealed muffled heart sounds, an S3 gallop rhythm, a palpable femoral pulse, weak peripheral pulses, and mild hepatomegaly (2 cm below the right costal margin).

The laboratory workup is given in Table 1.

**Table 1:** Laboratory workup CPK, creatine phosphokinase, CK-MB, creatine kinase-myocardial band, CRP, C-reactive protein, WBC, white blood cells, HB, hemoglobin, AST aspartate transferase, ALT, alanine aminotransferase, BUN, blood urea nitrogen

Parameter	Obtained Value	Reference Range
Troponin I	Negative	<0.03 ng/mL
CPK	201.5	30-200 U/L
CK-MB	67.1	<5 ng/mL
C-reactive protein	0.9	<0.8 mg/dL
WBC	12.76	6-17 (× 10^3^/mL)
HB	9.8	10.5-12.0 (g/dL)
Platelet	340	150-350 (×10^3^/mL)
AST	64	20-60 U/L
ALT	27	12-45 U/L
Glucose, serum	31 mg/dL	60-100 mg/dL
BUN	22	2-20 mg/dL
Creatinine	0.32	0.3-0.7 mg/dL
Potassium	4.65	4.1-5.3 mEq/L
Sodium	136	135-145 mEq/L
Ammonia	29.7	29-150 mcg/dL

A CK-MB level of 67 ng/mL in a child with suspected myocarditis is clinically significant and supports the presence of acute myocardial injury. It strengthens the diagnostic impression when correlated with symptoms and imaging findings. However, caution is warranted due to its limited specificity, and further evaluation, including troponin levels and serial echocardiography, is essential for determining prognosis and guiding treatment.

A peripheral anaerobic blood culture was drawn, and after the incubation time, it showed no significant bacterial growth.

**Figure 1:**
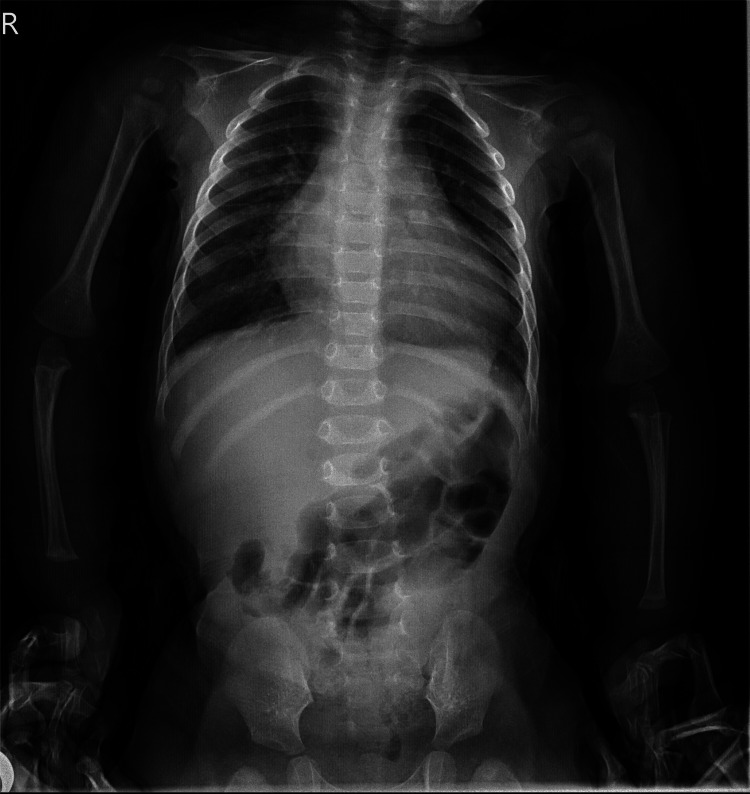
Anteroposterior chest radiograph showing marked cardiomegaly with an increased cardiothoracic ratio, reflecting significant cardiac enlargement.

**Figure 2:**
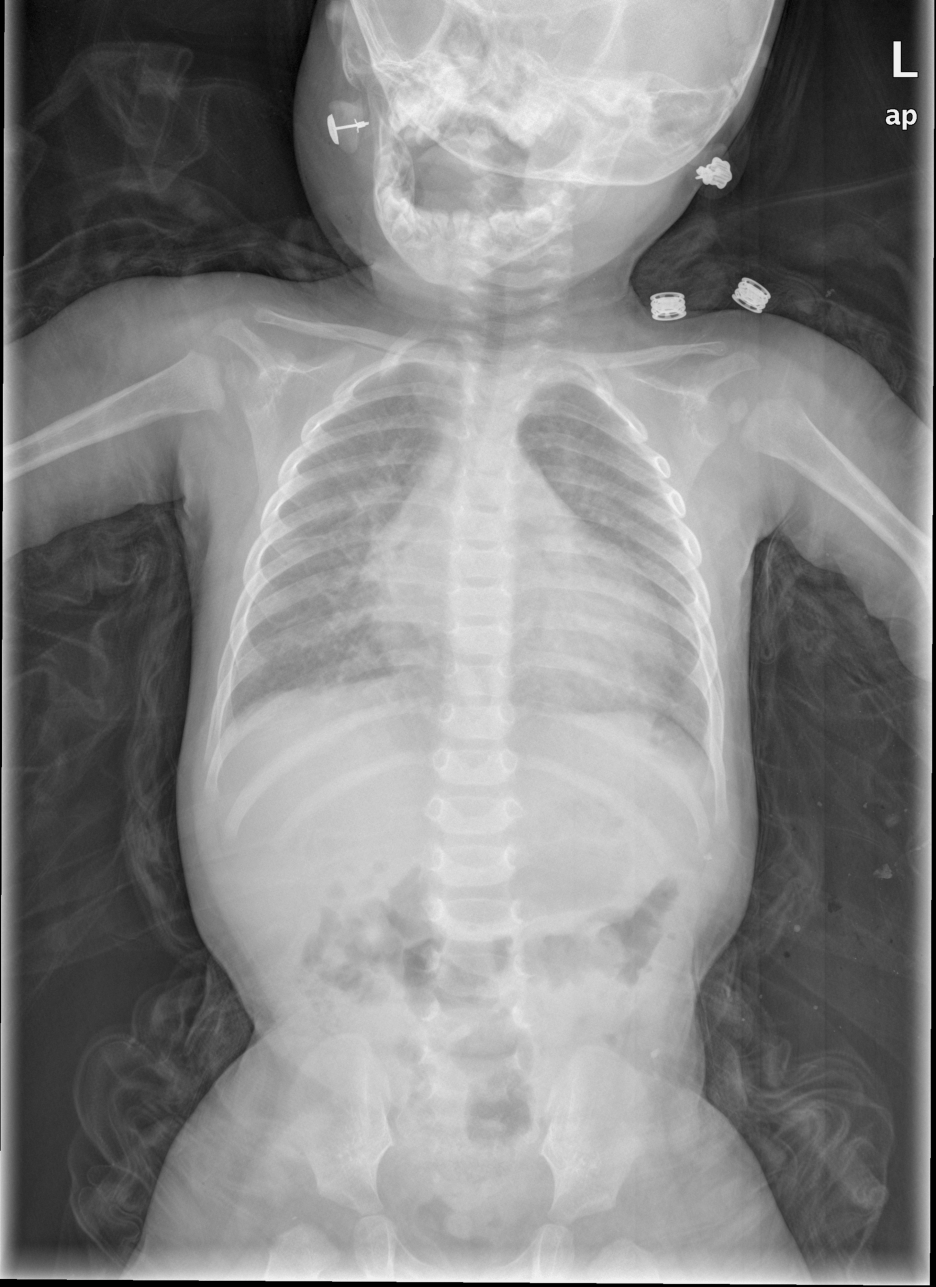
Anteroposterior chest radiograph showing cardiomegaly with increased cardiothoracic ratio and mild pulmonary congestion. These findings are consistent with acute cardiac dysfunction.

The initial chest X-ray was performed upon arrival at the emergency room (Figure 1). The next day, the chest X-ray showed pulmonary edema (Figure 2).

Echocardiography on admission demonstrated moderate left and right ventricular impairment, normal aortic arch and branches, normal pulmonary arteries with a fractional shortening of 17-19% normal approximately (28-42%), moderate tricuspid regurgitation, a mean pressure gradient (PG) of 30 mmHg, mild mitral regurgitation, no vegetation, and minimal pericardial effusion.

Supplemental oxygen was administered via a Vapotherm high-flow nasal cannula. The hypoglycemia glucose level of 31 mg/dL was managed with an intravenous bolus of 10% dextrose at 2 mL/kg, followed by a maintenance infusion of 5% dextrose in 0.9% saline. To provide inotropic support and enhance myocardial contractility, dopamine was initiated at a rate of 5-10 mcg/kg/min during the first five days of hospitalization. Inotropic vasopressor agents, such as epinephrine, were considered potential interventions in the event of severe hypotension or cardiogenic shock; however, the patient remained hemodynamically stable and did not require additional agents.

With the assumption of immune-mediated myocardial damage, likely due to a preceding viral infection that was based on clinical context alone, 2 grams/kg of IVIG was administered over 48 hours alongside methylprednisolone therapy. For the IVIG infusion, the following protocol was adopted: 2.25 mL/hr during the first hour, 4.5 mL/hr in the second hour, and 7 mL/hr for the remainder of the infusion.

The patient demonstrated marked clinical improvement following therapy with methylprednisolone, administered according to the treatment protocol tapering schedule outlined in Table 2.

**Table 2:** Methylprednisolone dose and days of treatment

Treatment day	Dose (methylprednisolone equivalent dose)
Days 1–3	10 mg/kg daily (maximum 750 mg)
Days 4–7	2 mg/kg daily
Week 2	1 mg/kg daily
Week 3	0.8 mg/kg daily
Week 4	0.7 mg/kg daily
Week 5	0.6 mg/kg daily
Week 6	0.5 mg/kg daily
Week 7	0.4 mg/kg daily
Week 8	0.3 mg/kg daily
Week 9	0.2 mg/kg daily
Week 10	0.1 mg/kg daily
Week 11	0.05 mg/kg daily

Table 3 presents the follow-up echocardiogram findings throughout the course of treatment. By day 7 post-intervention, left ventricular function showed significant improvement, with the ejection fraction rising to 29%. However, some fluctuation in fractional shortening and persistent ventricular dilation were observed, indicating ongoing myocardial remodeling. The patient was successfully weaned off inotropic support by day 5 and discharged on day 10 with oral carvedilol (1.8 mg once daily) and captopril (2.25 mg every 12 hours) to support cardiac remodeling after cessation of inotropic therapy. At the five-month follow-up, the patient remained asymptomatic with normal biomarker levels.

**Table 3:** Echocardiographic findings and clinical course GII, grade 2; LVFS, left ventricular fractional shortening; LVEDd, left ventricular end-diastolic dimension; LVESd, left ventricular end-systolic diameter; MR, mitral regurgitation; PG, pressure gradient; TR, tricuspid regurgitation

Parameter	November 11, 2024 (day of admission)	November 17, 2024	December 23, 2024	January 21, 2025	April 15, 2025
LVFS (fractional shortening)	17-19%	29%	21%	23%	23%
LVEDd (cm)	3.4	3.6	3.1	3.1	3.1
LVESd (cm)	NR	2.4	2.8	2.4	2.4
Pericardial effusion	Minimal	None	None	None	None
Valvular regurgitation	MR: mild; TR: moderate	MR: trace; TR: trace	MR: GII+	MR: GII+	MR: GII+; TR: trace
TR gradient (PG) (mmHg)	30	30	27	27	27
Left and right ventricular function	Mild-to-moderate impairment	Improving (near-normal)	Impaired and dilated	Impaired and dilated	Impaired and dilated

## Discussion

Pediatric myocarditis is a rare but potentially life-threatening condition, characterized by diverse clinical presentations that can complicate both diagnosis and management. Coxsackievirus and adenovirus are among the most common etiological agents. In this report, we present the case of a previously healthy 12-month-old female infant who developed acute myocarditis following an upper respiratory tract infection, a presentation consistent with post-viral pathogenesis. Confirmatory viral diagnostics could not be performed during the study because our laboratory did not have access to brain natriuretic peptide (BNP), lactate testing, or PCR assays for adenovirus and Coxsackievirus. Therefore, the diagnosis was based solely on the clinical context.

Myocarditis, particularly in children, presents a multitude of complications due to its nonspecific signs and symptoms, which can range from mild fatigue to severe heart failure [3]. Pediatric cases often present with respiratory distress, a symptom that typically raises concern [4]. In the case described, the combination of fever, lethargy, and respiratory distress, alongside laboratory findings showing elevated cardiac enzymes and echocardiographic evidence of biventricular dysfunction, provided sufficient grounds for a clinical diagnosis without the need for invasive procedures such as EMB.

Although EMB has traditionally been considered the gold standard, its role is contested, particularly due to limitations highlighted by the Dallas criteria. These include procedural risks, sampling errors, and challenges in obtaining sterile vascular access in young children [5,6]. As a result, noninvasive modalities such as echocardiography and CMR - guided by the updated Lake Louise Criteria - have become essential tools in diagnosing myocarditis [8].

Immunomodulatory therapy for myocarditis relies primarily on corticosteroids, targeting the inflammatory cascade that leads to myocardial tissue injury in cases of inflammatory cardiomyopathy [1]. However, the use of IVIG remains controversial. While some evidence suggests that IVIG may reduce myocardial inflammation due to its immunomodulatory properties, robust data from controlled trials are lacking. Systematic reviews indicate that IVIG may improve ventricular function in pediatric patients, though a consistent mortality benefit has not been demonstrated [12]. Nonetheless, some experts advocate for IVIG use in selected cases involving autoimmune-mediated myocardial damage [3].

In this case, the patient received high-dose methylprednisolone and IVIG concurrently, resulting in significant clinical improvement. A follow-up echocardiogram revealed that the patient’s left ventricular ejection fraction had increased from 17-19% to 29% within a week. These findings align with previous studies suggesting that early IVIG administration can facilitate rapid and clinically meaningful recovery in appropriately selected cases [13]. Despite mild residual valvular regurgitation at follow-up, the normalization of ventricular function and the absence of pericardial effusion were encouraging indicators of recovery.

However, myocarditis carries a long-term risk of progression to dilated cardiomyopathy (DCM) and chronic heart failure, particularly in cases with persistent ventricular dysfunction [4,5]. In the present case, one of the authors continued to monitor the patient via echocardiography and observed mild mitral regurgitation and ventricular dilation - early warning signs necessitating close long-term follow-up. While some patients experience full recovery, a notable proportion may develop chronic myocardial remodeling despite initial clinical improvement [13].

We advocate for the early use of IVIG in combination with corticosteroids in pediatric myocarditis. However, more multicenter studies and randomized controlled trials are needed to establish definitive treatment guidelines [14]. A significant barrier in resource-limited settings is the lack of advanced diagnostic equipment. In such environments, the adoption of intuitive, noninvasive strategies using basic imaging tools is both practical and essential.

This case underscores the importance of initiating dual immunomodulatory therapy as early as possible in the treatment of pediatric myocarditis. The favorable clinical course observed suggests that combined IVIG and corticosteroid treatment may be a viable approach to improve outcomes, especially in settings where advanced heart failure therapies are not readily available. Continued research and collaborative efforts are critical to refining diagnostic criteria and optimizing treatment strategies for this complex and life-threatening condition.

## Conclusions

While marked clinical improvement was observed in this case following combined treatment with IVIG and methylprednisolone, it is important to acknowledge that this is a single case report. As such, causality cannot be definitively established, and the findings may not be generalizable without support from larger, controlled clinical studies.

This case involved a 12-month-old female infant; however, the therapeutic approach should not be universally applied across all pediatric age groups without further clarification. Future research should investigate age-specific responses, including those in infants, young children, and adolescents, to ensure that treatment strategies are appropriately tailored to each subgroup. The synergistic immunomodulatory effects of IVIG and systemic corticosteroids are of particular interest in pediatric myocarditis, especially in cases of suspected immune-mediated inflammation or viral myocarditis accompanied by a heightened inflammatory response. IVIG is believed to modulate the immune system, while corticosteroids offer potent anti-inflammatory action that may reduce myocardial injury. In the present case, dual immunotherapy was associated with significant clinical improvement.

Nevertheless, due to the heterogeneity of pediatric myocarditis and the limited availability of pediatric-specific data, there remains a critical need to refine and validate combination immunotherapy strategies. Key areas for future investigation include the determination of optimal dosing protocols, patient selection criteria, and long-term safety and efficacy outcomes. Additionally, consideration must be given to the safety profile, cost, and accessibility of such treatments, particularly in low-resource settings. To advance the field, we advocate for multicenter collaboration, the development of evidence-based clinical guidelines, and the implementation of prospective, randomized clinical trials to establish the role of dual immunotherapy in the management of pediatric myocarditis.
